# Effects of changing the head coach on soccer team’s performance: A systematic review

**DOI:** 10.5114/biolsport.2024.131816

**Published:** 2023-04-10

**Authors:** Honorato Sousa, Filipe Manuel Clemente, Élvio R. Gouveia, Adam Field, Hugo Borges Sarmento

**Affiliations:** 1 University of Coimbra, Research Unit for Sport and Physical Activity. Faculty of Sport Sciences and Physical Education, Coimbra, Portugal; 2 Escola Superior Desporto e Lazer, Instituto Politécnico de Viana do Castelo, Rua Escola Industrial e Comercial de Nun’Álvares, 4900-347 Viana do Castelo, Portugal; 3 Instituto de Telecomunicações, Delegação da Covilhã, Lisboa 1049-001, Portugal; 4 Department of Physical Education and Sport, University of Madeira, Funchal, Portugal; 5 LARSyS, Interactive Technologies Institute, Funchal, Portugal; 6 Research Centre for Musculoskeletal Science and Sports Medicine, Department of Sport and Exercise Science, Manchester Metropolitan University, Manchester, UK

**Keywords:** Manager, Football, Replacement, Effects, Athletic performance

## Abstract

The aim of this review was to identify and synthesise the most significant literature addressing the effects of changing the HC on soccer team’s performance, identifying the most frequently researched outcomes and characterizing their methodologies. A systematic review of PubMed, Scopus, Web of Science and SPORTDiscus databases was performed according to the Preferred Reporting Items for Systematic Reviews and Meta-Analyses (PRISMA, 2020) guidelines. The following keywords and synonyms were entered in various combinations in the title, abstract or keywords: “football*” OR soccer AND “coach*” OR “manager*” AND “replacement” OR “turnover” OR “substitution*” OR “change”. Solely original articles written in English that contained data about the effects of the change in the HC on performance in professional male soccer were included. A total of 94 titles were identified, of which 24 met the eligibility criteria. The quality of the studies was considered excellent. The most common topic of analysis was the effects of the HC on match outcomes (i.e., win, draw, loss, points won, goals average). Some studies suggest that the HCs dismissal has small but positive impacts on a team’s short-term performance, while other findings suggest that little-to-no impact is observed following HC departure. The dismissal of the HC does not guarantee increase success of an underperforming team. Some studies suggest that hiring an appropriate coach could positively affect match performance in the short-term. Due to limited variations in study designs, further research is needed before robust conclusions can be drawn.

## INTRODUCTION

Soccer is a sport with enormous global popularity, mobilizing large masses and with great economic impact. This context tends to create increased pressure on professional clubs, which seek to optimize performance and obtain better results. In the presence of failure, the common tendency is to replace the HC. This replacement usually occurs when a team has a run of negative results over a consecutive period of games, trying to break the negative sequence [1–3].

Contemporary data indicates that the HC turnover rate in professional soccer is high [4–6]. For instance, out of 120 HCs in the top Brazilian soccer league between 2012 and 2017, 87 coaches were dismissed during a season, making the median appointment of a HC around 16 games [5]. Withing the “Big Five” European soccer leagues, only during the 2017/18 season, there were 55 coaching changes [6]. The coach’s dismissal usually occurs in response to reductions in a team’s performance, typically across a period of two months or twenty games [7]. Bryson et al. [8] report that dismissals in midseason peak when some leagues have a winter break, suggesting that there are within-season periods where dismissals are more likely to occur.

Even though sacking the HC from elite soccer clubs while the season is in progress could cause substantial disturbances in player and team stability, organizational growth, and financial safety [9], there appears to be three reasons as to why these dismissals occur. These factors relate to intentions to 1) improve performance due to recent match results and unsatisfactory position in the league, 2) increase the motivation of the players in pre- and post-game contexts, and (3) key stakeholder, sponsors and fans dissatisfaction [10–12].

The HC is the central individual accountable for the teams’ results, irrespective of the sport [13–14], with HCs influencing the team’s performance, and players’ mental wellbeing and physical loads [15]. Some scientific evidence revealed that coach encouragement can be a helpful tool for manipulating the internal and external training load [16–17]. The style of leadership of the HC can also be related with the occurrence of severe injuries [18]. Therefore, match results can be influenced by many variables, with the HC playing a key role in the planning and executing the tactical, technical, physical, and psychological aspects of a soccer team, being the major responsible for the training and competing process.

A review of the literature reveals that the effect of changing coaches has focused mainly on the effects on results, namely victories, defeats, draws, goals scored/conceded and position variation in the league table [3, 9, 19–20]. The scientific evidence is scarce regarding studies that analyse the effects of changing HCs on the locomotor responses of players [21], with some indicating that the change did not cause significant physical changes [22–23]. To the best of our knowledge, only the study of Guerrero-Calderon et al. [24], has verified how coach turnover affects physical responses in players and teams’ performances, both in competition and training, presenting some reductions in both contexts with the arrival of a new coach. Additionally, Radzimiński et al. [25], compared match outcome and physical match performance between sacked coaches and new coaches with reference to a control group (teams that did not replace the coach during 3 consecutive seasons). The results suggested that the change may result in short-term improvement in team results and physical performance, however the effect tends to disappear after 5 games following the arrival of the new coach.

Thus, there seem to be insufficiencies in the knowledge related to the performance variables (and its variability) of a professional soccer player when the HC is changed, leading to an increase in recent years in the number of publications on performance and training variables in players caused by the change of coaches [3, 8, 20, 24–25]. This knowledge can provide additional information about a better adaptation to the coach turnover process (i.e., to the “methodological whiplash” and not just “psychological”), in addition to the usefulness in monitoring loads, understanding mood states and well-being of the players, when replacing coaches [24].

Analysing the available literature in relation to the effects of the HC dismissal on teams’ performance in variables related to teams’ performance (e.g., number of wins, losses, draws, points won, variation in league table and number of goals scored/allowed) and players locomotor responses (e.g., total distance covered, distance per minute, high speed running, max speed, accelerations, decelerations and player workload), might support with stakeholder decision-making processes. Understanding the effects of HC replacement might result in longer-term success for soccer clubs through the support of data-driven decision-making. Therefore, the aim of this article was to systematically review and organize the literature on the effects of changing the HC on professional male soccer teams’ performance. This research was also undertaken with a view to identifying frequently researched effects and outcomes, characterising the methodologies, and systematising the evolution of the related research to provide information for clubs’ authorities relating to the consequences of replacing the HC.

## MATERIALS AND METHODS

### Search Strategy: Databases and Inclusion Criteria

This systematic review was reported in accordance with the PRISMA guidelines [26]. Electronic databases (PubMed, Scopus, Web of Science and SPORTDiscus) were searched for relevant publications on 10^th^ June 2023. Searches included the following keywords “football*” OR “soccer” AND “coach*” OR “manager*” AND “replacement” OR “turnover” OR “substitution*” OR “change”. Reference managing software (EndNote X9, Thomson Reuters©, New York, NY, USA) was used for managing records.

The inclusion and exclusion criteria can be found in Table 1.

The reference lists of retrieved studies were also reviewed to identify potentially eligible studies not captured by electronic searches. The retrieved records (namely titles and abstracts) were independently screened by two authors (HS and FMC). The same authors also independently screened the full texts. Disagreements between the two authors were deliberated in a collaborative reanalysis. In the case of no unanimity being reached, a third author (HBS) made the final decision. Where doubts occurred in the selection process, all co-authors shared opinions until consensus was achieved.

**TABLE 1:** Eligibility criteria.

	Inclusion Criteria	Exclusion Criteria
Population	Professional male soccer players irrespective of the competitive level.	Sports other than soccer (e.g., futsal, beach football, American football, Australian football), female and youth players, amateur senior teams.

Exposure	Exposure to the change of the head-coach independent of duration.	Not controlled for exposure to the change of the head-coach

Comparator	Not mandatory. If possible: Compared with teams that were in same context as the teams that dismissed the HC, but the sacking did not occur.

Outcome	Locomotor performance (e.g., total distance covered, distance per minute, high speed running, maximum running speed, accelerations, decelerations, and total workload), sports results (e.g., win, lose, draw).	Other measures not related to athletic and match performance, training load, and wellness measures (e.g., injury occurrence).

Other	Only original studies (restricted to articles written in English), published no later than 2010. Only articles that circumscribe dismissals during the course of the season.	Articles not written in English, abstracts, proceedings, book chapters, letters, and other non-original studies.

The data extraction procedure was primarily executed by the lead author (HS) and was verified by two co-authors (FMC and ERG) to corroborate the accuracy and specifics of the data. An adapted Microsoft^®^ Excel datasheet was generated and used to include the data and the main information. In the absence of key data, the primary author (HS) directly contacted the corresponding author by email and/or ResearchGate to acquire the information.

### Quality of the Studies and Extraction of Data

As recommended in Faber et al. [27], the methodological assessment process was accessed using the Law et al. [28] scale for quantitative studies (counting 16 items) and was performed by two authors (HS and FMC). The studies were assessed to determine whether they included the following 16 items: the objective (item 1), relevance of background literature (item 2), appropriateness of the study design (item 3), sample included (items 4 and 5), informed consent procedure (item 6), outcome measures (item 7), validity of measures (item 8), significance of results (item 10), analysis (item 11), clinical importance (item 12), description of drop-outs (item 13), conclusion (item 14), practical implications (item 15) and limitations (item 16). Item 9 (details of the intervention procedure) were not applicable because none of the studies included interventions.

For each item, the quality was rated as 1 (meets criteria), 0 (does not meet the criteria fully), or NA (not applicable). A final score expressed as a percentage for each study (Faber et al. 2016). The final score corresponded to the sum of every score in each article, divided by the total number of scored items for that specific research design. We adopted the classifications of Faber et al. (2016) and Te Wierike et al. [29] and classified the articles as (1) low methodological quality – with a score ≤ 50%; (2) good methodological quality – score between 51 and 75%; and (3) excellent methodological quality – with a score > 75%. The quality analyses were undertaken by the lead author (HS). Disagreements were subsequently solved via diligent debates between two of the authors (HS, FMC). For organizational purposes, the studies were classified into categories according to the major research topics emerged from the content analysis.

## RESULTS

### Search, Selection, and Inclusion of Publications

**FIG. 1:**
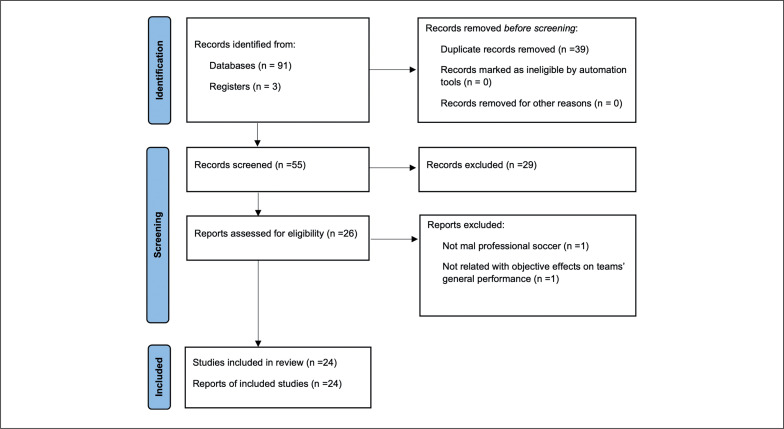
PRISMA flow diagram.

The initial searches retrieved 91 titles in the databases (PubMed – 22 articles; Scopus – 24 articles; Web of Science – 27 articles, and SPORTDiscus – 18 articles) and three more articles from Registers. Any duplicates (39 references) were eliminated either automatically or manually. The remaining 55 articles were then assessed for relevance based on their titles and abstracts, resulting in 29 studies being removed from the database. The full text of the remaining 26 articles were examined; 2 were excluded because they did not meet the inclusion criteria. At the end of the screening process, 24 articles were selected for detailed reading and analysis (Figure 1). One study was excluded (n = 1) because of their shortage of relevance with objective effects on teams’ general performance (n = 1). The other study (n = 1) was excluded because it contained data not relevant to male professional soccer. The articles considered in this review were published before 2010, with approximately 60 % published in the last 6 years (i.e., 2016 to 2022).

### Quality of the Studies

Concerning the quality of studies included, the most significant results were that: (1) the mean score for the studies included in this review was 84.8%; (2) All studies scored above 50%; (3) three studies achieved 100%; (4) only two studies scored between 51 and 75%; and (5) twenty-one studies reached an overall rating above 75%.

Tabela qualidade dos estudos – material suplementar.

### General Description of the Studies

Twenty-four studies were included in the qualitative analyses. All analysed studies were carried out in adult male professional soccer. The majority of studies (*n* = 22) were conducted in teams competing in the English Premier League, Spanish La Liga, German 1. Bundesliga, Italian Serie A, French Ligue 1, Portuguese Super Liga, Dutch First Division and Belgium First Division). The remaining two studies, reported data from the Polish and Argentinian first divisions.

Results are presented in Table 2. Match results or points won were included in thirteen studies [3, 7, 8, 11, 12, 30, 31, 32, 33, 34, 35, 36, 37]. Four other studies combined different sub-topics, such as the points won with the number of goals scored and conceded [23, 38, 39, 40]. One other study used the points won to compare team’s performance when coached by the new and the old HC, also scrutinizing the effect of the change with some coach related factors, such as coaching experience or if the new coach being a former elite player could influence the performance [6].

**TABLE 2:** Studies that analyze the impact of HC replacement using the team’s sports performance results and physical performance.

Study	Aim	Sample	Performance Measurement	Study Design	Findings	Quality Score (%)
Radzimiński et al. [25]	To examine the physical match performance and match outcome before and after coach turnover compared with a control group containing coaches working continuously for 3 consecutive seasons.	325 performances of teams led by dismissed HCs, 313 of teams led by new HCs, and 580 of teams led by unchanged HCs in first Polish League.	Average number of collected points, total distance, total distance per minute, high-speed running, sprinting and number of high-intensity runs.	Split into periods when the team was led by a coach who was going to be dismissed (DC) and into a period when the team was led by a new coach (NC). For each HC, a group of the last 15 matches was analysed. The first 15 games of NC were taken into consideration.	The study reported that mid-season HC change may result in short-term improvement in team results and physical match performance. However, this effect disappears after a period of approximately 5 games.	92.8%

Bryson et al. [8]	To compare the performance of teams after they have sacked their HC with spells where the HC remains in the job.	Games from the top two divisions of four major European soccer leagues (France, Germany, Italy, and Spain) over the period 2001/02 to 2014/15. 1,327 HC dismissals.	Number of points won by teams.	Included variables such as opposition form, measured by the opponent’s league positions, and home advantage, measured by the proportion of home games over the follow up period. Also incorporate a measure of surprise which is the difference between actual and expected performance.	Teams who fire their HC experience small but statistically significant improvements in team performance, although this positive impact is confined to circumstances in which a team holds onto the new HC having sacked the previous coach.	85.7%

Guerrero-Calderon et al. [24]	To analyse the locomotion and metabolic responses of professional players during the four weeks before and after the change of the HC.	Training and match data across 3 professional soccer teams belonging to the top three competitive standards of Spanish soccer have been analysed (*n* = 1189 events).	Training and match load data.	Training and match load data were analysed separately by HC to compare data between them. To ascertain the effect of the HCC, the individual physical and metabolic responses of players were analysed during 4 weeks before and 4 weeks after the coach dismissal during the competitive period.	Players modify their behaviour in relation to physical performance after the incorporation of a new HC due to the dismissal of the previous at mid-season. In training, players showed greater high-intensity activity with the previous HC rather than with the new one. A similar performance between HCs was found in the competition	92.8%
Gómez et al. [6]	To compare team’s performance when coached by new and old HCs. To investigate the impact of a HC change on team’s performance according to coach and club related factors.	411 mid-season coaching changes from the 2010–11 season to the 20017–18 season in the Spanish La Liga (n = 85), French Ligue 1 (n = 51), English Premier League (n = 79), German Bundesliga (n = 82) and Italian Serie A (n = 109).	Number of points won by teams.	Linear regression analyses were estimated to determine the winning profile of a new coach. The dependent variable was the points awarded over 1, 2, 3, 4, 5, 10, 15 or 20 matches following the HCC.	Team’s short-term performance was improved significantly with a change to a new coach with this impact declining in the longer term (> 10 matches). The winning effect due to the new coach was independent of coach-related factors such as coaching experience or the new coach being a former elite player.	100%

Scelles and Llorca [20]	To examine the effectiveness of dismissing the HC.	In-season leader dismissals and team performance data from games played in the French men’s soccer Ligue 1 over the 1994–2016 period (n = 4918 observations, out of the 7990 games played).	Throughout goal difference, position at the table and competitor position.	Used a dummy variable based on the evolution of the team position in the table over the last three games leading to the leader change. Calculated the cumulative difference in the team positions over the same three matchdays between two seasons, one with an HCC after the third matchday and another without an HCC.	Performance improves after a HC dismissal. At the same time, performance improves after a HC dismissal that does not happen (control group), i.e., the HC is not dismissed in a situation when the HC has been dismissed at another time.	100%
Zart and Güllich [3]	To investigate effects of in-season HC changes on the subsequent team performance.	149 HCCs’ and 3,960 games between 2010–19 in men’s English, German, and Spanish premier soccer leagues.	Points achieved relative to expected points for each game.	Preand post-HC change short, medium, and long-term performance (points achieved relative to expected points for each game, taking account of opponents’ current strength and whether a game was played at home or away).	Performance showed an instant, strong improvement in the first post-HC change game. The data suggests positive short, medium, and long-term HC change effects at the highest professional soccer level.	100%

Arrondel, Duhautois and Zimmer [12]	To analyse the impact of within-season HC change on club performance.	4 seasons with 18 teams and 16 seasons with 20 teams (392 teams-seasons observations and 7304 games) from French Ligue 1.	Number of points won by teams.	Used an empirical method that takes observable differences between clubs into account (through exact matching) and corrects for unobserved characteristics (through difference-indifferences).	The overall effects of a HC change on team performance are insignificant, except in the short term where they are positive and statistically significant. Between home and away games, the effect is only positive and significant for home games.	78.5%

Rocaboy and Pavlik [2]	To identify why professional soccer clubs, replace their HC and investigate the effect of HC dismissal on team performance.	English Premier League and the Ligue 1 fixtures for the 2015/16 and 2016/17 seasons. 31 HCC, 18 in the French Ligue 1 and 13 in the English Premier League.	Final league positions of the teams (considering the total wage costs by team) and points won (number of wins, draws and losses).	Used a Monte Carlo simulation (method to produce probability distributions for the final league positions of the teams – this one based on the total wage costs) and compared the final league position with the expected final league position.	HC dismissal results on a drop in the average expected performance compared with the performance expectations at the beginning of the season. Dismissing a coach may enhance performance only if the team under-performed before the dismissal.	85.7%

Scelles and Llorca [40]	To analyse the impact of an HC change on team performance in men’s soccer.	3,600 games (94 team-seasons) in French Ligue 1 over the 2000-2016 period.	Points won (considering whether the game was won or not) and the goal difference.	Explanatory variables were used, such as: - home Advantage; the position of the team prior to the Game; the position of the opponent prior to the game; the position of the opponent at the end of the previous season.	An HC change may have a positive impact on team performance, supporting the idea of considering this option when looking for some ways to improve performance.	92.8%

Argentieri, Canova and Manera [41]	To identify the effects of replacing a HC at mid-season due to poor team performance.	38 Serie A games per team and season (over ten seasons – 2007/2008 to 2016/2017).	Player marks given by specialized newspaper and points made by a team in a season.	From the marks, it was calculated an average that served as an indicator for overall team performance in the matches. With the total points obtained by teams at the end of each season it was showed the outcome obtained by each team.	Very low positive impact of the HC change in the short term but a significant negative impact in the long term.	78.5%

Wilson, Plumley and Flint [19]	To examine the effect of HC change in the English soccer industry and explain the association between HC change and organizational performance.	2,816 matches, 525 instances of managerial change in all four main English Football Leagues (EFLs).	Total number of points obtained by a team during an entire season against the total number of matches played by any given team.	The average points per match of those clubs that have experienced managerial change during the last 16 years were analysed. Used league position as an additional proxy to measure sporting success.	The results show significant differences in all four EFLs when considering teams who make a HC change and those who do not. A HC change is more beneficial for clubs in the bottom half of the league.	85.7%

Besters,Van Ours and Van Tuijl [37]	To analyse the performance effects of in-season HC change.	84 in-season managerial changes in English Premier League soccer between 2000/2001 and 2014/2015 seasons, 5700 matches in total.	Number of points won by teams.	Focused on two variables: a treatment group, where the HC had been replaced and measuring the effect of this HCC on the performance; a control group, where the ‘hypothetical’ change has taken place, to measure the counterfactual effect on the performance.	Some HC changes are successful, while others are counterproductive. On average, performance does not improve following a HCC.	78.5%

Desai, Lockett and Paton [35]	To examine the effects of HC succession on post succession organizational performance.	19 newly appointed leaders in the English Premier League (1996–2010) and 4,452 match observations.	Number of points won by teams.	Different models were analysed, such as: leader to leader entry (HC was directly hired from another organization where that person was a HC); HC domestic experience; HC foreign experience. For control variables, the number of games a leader was in position since the appointment; total number of seasons a leader has held a leader position prior to appointment were some of the used.	HC that moved directly from a HC position at another organization performed better. HCs with domestic top experience are associated with lower post succession performance, while HCs with foreign top coaching experience are associated with higher post succession performance.	85.7%

Muehlheusser, Schneemann and Sliwka [36]	To analyse the impact of HC on performance: the role of team heterogeneity (teams’ composition and player abilities)	Dismissals of HC in German Bundesliga 1 between seasons 1994-2010 and 4263 match observations.	Average number of points accumulated in the season up to the match under consideration (HCC).	All procedures were made after identifying teams with identical short-term results. Considered a team’s long-term performance history, measuring the average league position in the last three seasons and compared with the season performance at the moment of the HCC.	Significant positive impact but only for homogeneous teams. HCs dismissals showed that individual performance increases to a stronger extent in homogeneous teams.	78.5%

Van Ours and Van Tuijl [11]	To analyse the causes and consequences of in-season changes of the HC of association soccer teams.	Number of changes of the HC from the highest level of Dutch professional soccer during 14 successive seasons (2000/01-2013/14).	The difference between the actual number of points and the expected number of points, based on the odds of the bookmakers, number of points in recent matches.		Post-dismissal team performance is superior to pre-dismissal team performance. Team performance after “coach dismissals that did not happen” is also better than prior to dismissals that did not happen. There are no positive performance effects for a club dismissing the HC.	78.5%

Flint, Plumley and Wilson [34]	To examine whether HC change was beneficial in acquiring more points per match and whether HC change improved final league position.	A total of 38 league matches per 20 teams in 10 consecutive years/seasons (7600 games) in the English Premier League.	League points and league matches as the main proxy for the analysis and considered the average points per match obtained by each HC. League position before and after the change was considered.	For all analyses, the points per match and league position before HCC was compared with the rest of the season.	HC change led to an increase in points per match but did not necessarily lead to an improvement in final league position.	92.8%

Flores, Forrest and Tena [33]	To analyse the dismissals of soccer HC and their consequences.	420 within-season managerial changes in the top division of Argentine soccer between seasons 1986/87–2005/06 (7000 games).	Points per game.	Team performance was measured at the time of the HCC, in the form of points per game achieved in the preceding four matches and points per game achieved in the season to date, also considering the context of the fixture (away or home game).	There was a difference in impact from HC change according to whether home or away results were examined. The negative effects of change were easier to detect for away games. Dismissals tend to have a net adverse effect on the team, but the effect is perhaps moderated in home games.	78.5%

Maximiano [39]	To investigate whether replacing a HC improves teams’ performance.	Portuguese Super Liga seasons between 1999-2005, 3,672 match observations.	Match-level team performance data and a propensity score matching triple difference estimator (average points and average goals scored and conceded in the five matches before/after).	Used a control group with the matches of teams that did not fire the HC but that share similar observable characteristics and an identical pre-firing performance history with those whose HC was fired.	On average, teams perform better with the new HC: more wins, goals and concede fewer. These effects disappear when compared the improvement in performance with the teams that had similar performance and not fired the HC.	78.5%

González-Gómez, Picazo-Tadeo and Garcí-Rubio [31]	To evaluate the impact of a midseason, change of HC on the sporting performance of professional soccer teams	56 HCC in Spanish soccer teams that played in La Liga between seasons 2001/2002 to 2008/2009.	Throughout points per match (obtained at the end of the season and the extra matches played in other competitions).	Data envelopment analysis techniques were used to compare the performance of a group of teams that have changed their HC midseason to that group of teams that have stuck with the same HC for the entire season.	A mid-season change of HC improves sporting performance, but it does not allow to performas well as teams that have not changed HC halfway through the season.	71.4%

Heuer et al. [23]	To study if the HC change during the mid-season has an impact on the subsequent team performance and fitness levels.	14,018 games, starting in the season 1963/64 of the Bundesliga to the 2008/09 season.	Number of goals scored or conceded, and the points won (per match).	Used a statistical framework for accessing the team fitness based on the number of goals scored or conceded, also using the points won (per match) to evaluate teams’ overall performance.	Dismissing the HC within the season had basically no effect on the subsequent performance of a team. The HC can bring changes in the physical levels of the team, but these are diluted over time and return to values considered comparable or similar to the previous coach.	78.5%

Lago-Peñas [32]	To investigate the relationship between team performance and HC change over time.	HCCs in male soccer teams that played in the highest national division and the second division in Spain during the seasons from 1997–1998 to 2006–2007.	Percentage of points gained by teams in the matches 1, 2, 3, 5, 10, 15 or 20 prior and following to date of the HCC.	A comparison between the mean team performance levels with the old and a new HC was done over 1, 2, 3, 5, 10, 15 and 20 matches before and after the date of resignation.	No impact of HCC in the long term. The favourable short-term impact of an HCC is followed by continued gradual worsening of results. The HCC effect is non-existent when the comparison is done over 10, 15 or 20 matches before and after termination.	78.5%

Paola and Scoppa [38]	To study the effects of HC change on teams’ performance.	12 seasons of the major Italian soccer league ‘‘Serie A’’ (starting from 1997–1998 to ceded. 2008–2009), a total of 4,042 matches.	Points won (per match) and number of goals scored or conceded.	Estimated a number of regressions trying to consider the ‘‘Ashenfelter dip’’ by controlling for team fixed effects, team quality indicators, and lagged match results. Used a matching estimator to estimate the average treatment effect of the HCC, comparing teams that have changed with those hadn’t, with similar scenarios.	Changing the HC did not affect team performance, neither when considering as dependent variable the number of points per match nor when looking at the number of goals scored or conceded.	78.5%

Balduck, Buelens and Philippaerts [7]	To examine the short-term effects of midseason HC turnover on soccer team performance.	Game outcomes of Belgian male soccer teams from the first, second and third national divisions from the 1998/99 season to the 2002/03 season.	Points won (four-game point average divided by the seasonal average of points per game).	Define a short term as a span of 4 games prior to and next an HCC. A comparison was made by creating a control (HC not dismissal) and a turnover (HCC in teams with the same performance pattern) groups.	Results showed that changing the HC to improve short-term performance is not the most appropriate way to deal with a bad sequence of results. Teams with the same performance pattern as the turnover group significantly improved after a performance dip without changing HC.	71.4%

Hughes et al. [30]	To test for the short-term and long-term effects of HC change in comparison to the tenures of incumbent top HC.	Results of each competitive game played by every team (more than 5000 soccer matches) between English Premier League seasons of 1994 until 2004.	Points won/Match results (win/ draw/loss at home/away games).	Tested for the short-term and long-term effects of HC changes in comparison to the tenures of incumbent top HC. Divided the event window (HCC) into three periods. Ten matches after the change reflect the short term and 30 matches afterwards capture the long term.	Long incumbent tenures are associated with performance far above the average. When looking at change events, change in the short term leads to a brief reprieve in poor performance only for performance to deteriorate in the long term.	92.8%

An additional four studies considered the variation in the league table and the final league position, associating this sub-topic with the number of points won, the opponents’ strength or table position, the marks given by specialized newspapers to classify players’ individual performances, the context of the game (away or home game), and the total wage costs of the teams analysed [2, 19, 20, 34]. Three studies included the physiological component [23–25], with one of these studies analysing the physiological effects for both training and competition [24].

**FIG. 2:**
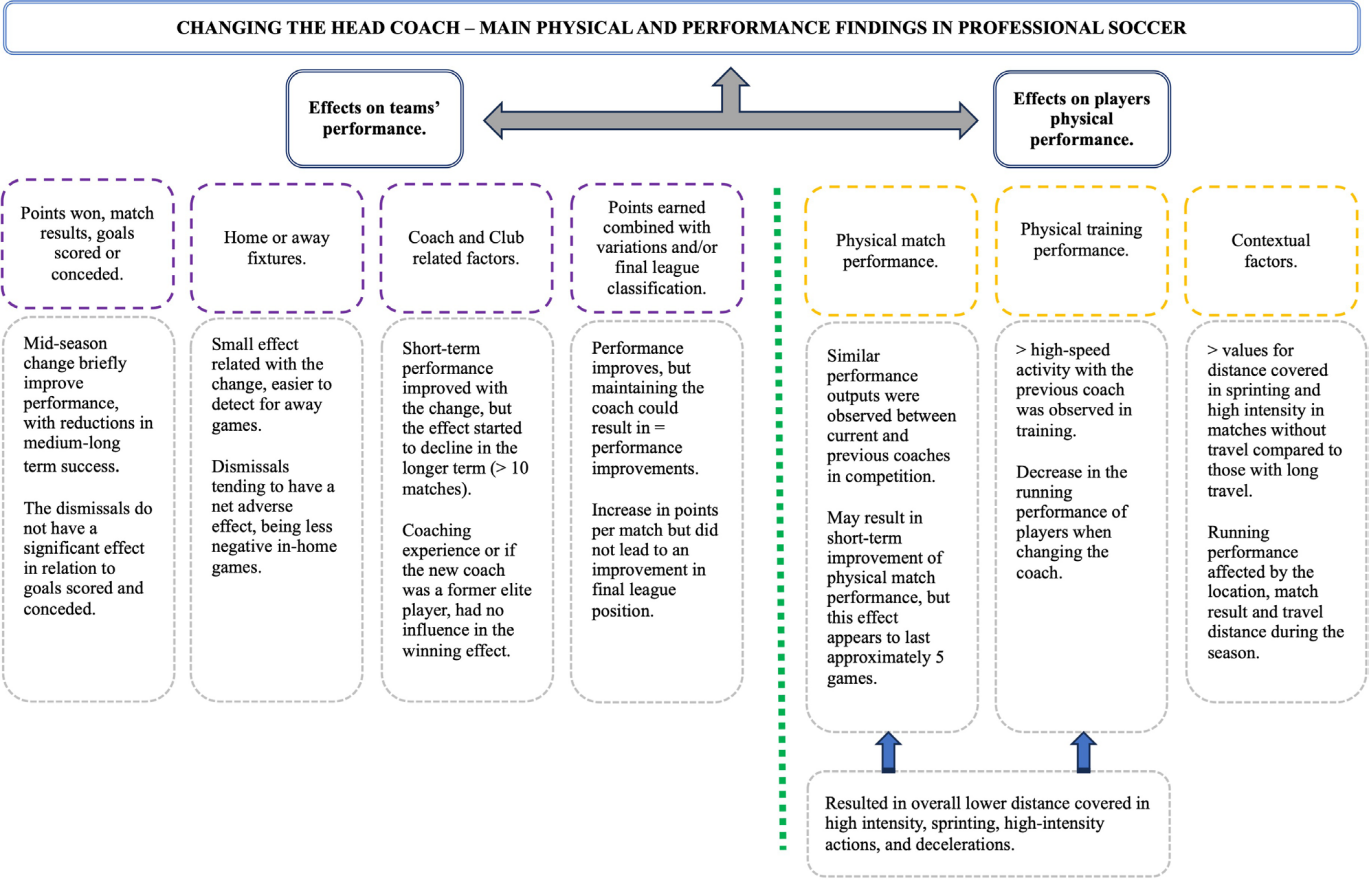
Main physical and performance findings in professional soccer.

Figure 2 makes a quick synthesis of the main variables under study and consequent key findings that the HCC produces in professional soccer.

## DISCUSSION

The aim of this article was to systematically review and organize the literature on the effects of changing the HC on team performance in professional male soccer. Two overarching outcomes were addressed, including, whether dismissal of the HC improves performance after multiple losses, and how performance is influenced with teams that remain with the same HC, despite multiple consecutive losses. The findings can be adopted to determine HC recruitment and retention strategies to inform decision-making processes on whether HCs should be dismissed or remain following periods of unfavorable results.

### Effects on teams’ performance

Discerning the effects of HC dismissal on performance between studies presented a key challenge given the variation in the performance outcomes. Outcomes varied between points obtained, goals scored and conceded, and league table position, which is often dependent on external factors (i.e., the success of other teams). In relation to studies that focused on the effects of the HC changes on points won or match results [8, 30, 31, 37, 39], a mid-season change of manager appears to briefly improve performance, but with reductions in medium-long term success. In this instance, teams that had not changed the HC performed better in the medium-to-long term that teams that changed the HC mid-season, possibly backing that long-term investment in the same HC is associated with longer term success versus mid-season management changes.

In the study of Gómez et al., [6], they compared team’s performance when coached by new and old HC, but also investigate the impact of a HC change on team’s performance according to coach and club related factors. The results showed that team’s short-term performance improved significantly with a change to a new HC, but the effect started to decline in the longer term (> 10 matches). Coaching experience or if the new HC was a former elite player, were some aspects analysed, and the winning effect was independent of this coach-related factors.

There were some studies that explored the impact of changing HC according to whether home or away results are examined [3, 12, 33]. These studies reported that there was a small effect related with the change of HCs, which was easier to detect for away games, with dismissals tending to have a net adverse effect on the team, with those effects being more moderated or less negative in-home games.

Considering the studies that focused on the points earned combined with variations and/or final league classification, some of the findings should be interpreted with caution. Scelles and Llorca [20], concluded that team performance improves after a HCs dismissal, however, maintaining the HC on the job could result in similar performance improvements. This evidence seems to support the idea of retaining the HC at the job, with gains related to sports performance and fewer costs associated with the non-dismissal of coaches and coaching stuff, in a mix of financial savings and promoting stability, time and space to develop the work of HC.

Flint, Plumley and Wilson [34] indicate that HC change led to an increase in points per match but did not necessarily lead to an improvement in final league position. Therefore, using league position as a measure of “performance” or “success” in this instance might not be entirely appropriate given that improvements in total points accumulation were observed. Rocaboy and Pavlik [2] showed that HC dismissal results in a drop in the average expected performance compared with the performance expectations at the beginning of the season. This suggests that the dismissal enhances performance solely if the team under-performed before the dismissal.

Two studies reported that the dismissal of a HC does not have a statistically significant effect on the subsequent performance of a team in relation to goals scored and conceded [23, 38], despite some research supporting that such changes seem to have a positive impact on team performance, supporting the idea of considering this option when looking for ways to improve performance [40]. Some studies also found that although performance improves after dismissal, the positive effects dissipate when compared with teams whereby the HC is not dismissed [20, 39]. This notion suggests that often premature dismissal can have detrimental implications for team performance and providing additional time for the HC might result in greater achievements, instead of resetting the process with another HC with the season ongoing.

Two studies used different and interestingly debatable variables for analyses. The first study assessed the difference between the actual number of points attained and the expected number of points based on the odds of the bookmaker [11]. The results demonstrated that post-dismissal team performance was superior to pre-dismissal team performance. Argentieri, Canova and Manera [41] analysed a combination of points won, average score of teams during a season and players ratings given by specialized newspapers. The results showed a small but positive impact of the HC change on short term success, but a significant negative impact longer term. However, the rigour the methodologies employed would appear questionable, given the subjectivity and biases associated with the financial and reputational conflicts of interest involved with bookmaker and journalist interpretations.

### Effects on players physical performance

It is important to understand that match outcome (wins, draws, defeats) could be influenced by the physical conditioning processes at individual soccer clubs. Konefal et al. [42] indicated the importance of parameters related to physical performance as highly predictive for the game result in professional soccer. Studies that analyse the modification of players’ behaviors in relation to physical performance after the replacement of the HC are scarce and seem to have debatable results.

A study reported that following the mid-season appointment of a new HC, similar performance outputs were observed between current and previous coaches in competition; however, greater highspeed activity with the previous HC was observed in training [24]. These findings are congruent with another study that showed a decrease in the running performance of players when changing the HC in training [21]. There are some variables that need to be considered to try to comprehend these outcomes. The methodological pattern of training can be different, with the two coaches presenting the players with different types of tasks or stimulus in training. For example, one coach applied exercises with large playing areas and, consequently, the players covering greater high-speed distances, and the new coach using drills based with reduced spaces, with more accelerations and decelerations. The new coaches’ initial sessions may have been designed to change tactics and playing styles to familiarize the players with new systems and ways of playing. Such sessions that incorporate more of a tactical-based impetus might not be intended to develop the physical component, and as such, might explain the current findings.

Other explanations might relate to the moment of the season when the new coach arrives, with emphasis on the management of training volumes and intensities. The mid-season transfer window can also alter HC practices with possible new signings being cautiously transitioned into new ways of playing. Chmura et al. [43] concluded that players’ physical performance tends to decrease after two-thirds of the season. Therefore, load and fatigue management are prioritized at the end of the season but with limited time to implement the new coaches’ principals, tactical behaviors, style of leadership and type of training. Also, a higher number of games in a small period can compromise the preparation of a team, and subsequently leave little time to train specific locomotor capabilities. Contextual factors, such as, the opponent quality, number of players injured and players coming back from injuries, as well as whether the game is a home or away game can also impact the preparation and physical performance of a team. Therefore, it is important that the findings of each of the studies are scrutinized before accurate inferences can be drawn.

Radzimiński et al. [25] found that mid-season replacement of the HC may result in short-term improvement of results and physical match performance, but that this effect appears to last for approximately 5 games. A separate investigation used [23], the goal difference as a proxy for the team’s fitness levels (i.e., the greater the goal different the greater the fitness). The findings indicate that the change of coach can bring changes that improve the team’s physical performance, but that these are diluted over time and return to values considered comparable to the previous coach. A possible explanation might relate to the commonsense theory that by dismissing the HC and bringing a new one, a psychological shock effect occurs. This shock effect might increase the players motivation instantly [32], but might not persist longer-term.

The gradual decline in physical match performance can be explained by the stabilization of the level of motivation related with the arrival of the new coach. When the psychological effect dissipates, the manager’s capability to lead the players is a critical influence in team performance [12]. Other variables such as specific management of volume and intensity of training, management of the injured players, or schedule overloads can be related to this phenomenon. This reiterates that the HC plays a key role in the motivation of the players and that innovative ways to increase the players desire should be sought by the HC.

Augusto et al. [21], stated that changing the HC during the season resulted in overall lower distance covered in high intensity, sprinting, high-intensity actions, and decelerations. Interestingly, the same study indicates that higher values for distance covered in sprinting and high intensity were found in matches without travel compared to those with long-travel, and overall, running performance was affected by the location, match outcome and distance traveled during the season. These variables related with specificities of the schedule and with the volatile environment of a soccer game, are important considerations to be deliberated, when reflecting about the midseason arrival of a new coach.

### Study Limitations

Some limitations should be considered according to the articles that were included in the present review. A limitation is that it only includes studies in English given the databases considered, thereby potentially overlooking other relevant publications written in other languages. Another aspect that was not considered, despite all articles included only reported in season changes of leadership, was the time during the season that the dismissal of the HCs happened. Aspects such as the congestion of games in the schedule at the time of the change of coaches, might be important since performance has shown to be influenced by fixture congested periods [44]. There were only three studies that assessed the physiological components in relation to HC dismissal. Only one of these studies utilized GPS tracking devices that allows you to collect detailed information, allowing the management of training processes and preparation for competition. Quantifying physical and physiological performance parameters using quantifiable metrics to assess the relationship with coach dismissal and performance might present future avenues of research.

### Practical Implications and future research

It has been a common practice in professional soccer to fire the HC at mid-season after a period of bad results, to try to reverse the negative streak. Understanding how this process takes place, and how it affects the behaviour and physical performance of the players, this type of information can support changes in tactical-technical and motivational strategies to be adopted during training and competition, optimizing load planning and avoid large variations that could negatively affect the players fitness by coaches and their technical staff. Also, this knowledge about the players physical behaviour could provide various information for clubs’ boards, helping to decide whether the dismissal can provide a positive stimulus, or contrariwise be a stress factor.

Some gaps in the available literature were found. First, there is currently a gap and a need for more longitudinal studies related to the analysis of the physiological component after the change of the coach. Structured studies are needed, able to circumscribe and control the largest number of variables that can affect the physical performance of players, to compare the influence and effects that a coach replacement can cause. In this specific situation of physical performance, there seems to be insufficient, or no studies, that could: 1) characterize the dynamics of training and competition in the locomotor performance indicators inherent to the process of replacing the HC; 2) analyse the dependence relationships of training and competition intensity considering the different coach profiles, 3) compare the players’ locomotor intensity indicators between a coach replacement process and a continuity process, and 4) verify if the locomotive variables related with the external load and the physical performance of professional football players in training and competition, can work as predictors for the retention or dismissal of professional soccer head coaches.

Another research gap identified during the process of searching for articles for this review, was the need for research on the psychological and environmental aspects impacting the performance of the players subject to coach change. Factors such as the effects caused on players who were playing (or not playing) with the previous coach, associating a comparative analysis with the physical performance of these same players, would be an interesting evaluation analysis in the context transition generated by the change of coach.

Even in the variable sports results, future research could consider involving outcomes related to the objective performance of the team, namely metrics such as number of shots, crosses or passes, percentage of successful and unsuccessful passes, number of ball recoveries, among others, to achieve an objective comparation related with raw game productivity between two or more different coaches with the same team.

## CONCLUSIONS

The findings of this systematic review suggests that dismissal of the HC does not always increase success of an underperforming team. However, some studies suggest that selecting and hiring an appropriate coach could positively affect match performance in professional soccer, particularly in the short-term provided that the coach has sufficient time for longitudinal strategies and philosophies to be implemented. Given the timely requirement for success in soccer, HC dismissals are frequent; but with the notion that persisting with a HC (albeit the correct one) longer-term could enhance a team’s performance, perhaps the model in which some board members at soccer clubs operate might need to be adjusted. These findings should be interpreted carefully since there are an abundance of external actors that can influence success when HCs are dismissed and hired, which may not be directly related to the HCs ability to improve performance. Further research should adopt more robust mixed methods study designs that statistically quantity associations with HC dismissal and variables of success, and qualitative analyses of stakeholder perceptions of success within soccer organisations.
